# Androgen and Luteinizing Hormone Stimulate the Function of Rat Immature Leydig Cells Through Different Transcription Signals

**DOI:** 10.3389/fendo.2021.599149

**Published:** 2021-03-17

**Authors:** Xiaoheng Li, Qiqi Zhu, Zina Wen, Kaimin Yuan, Zhijian Su, Yiyan Wang, Ying Zhong, Ren-Shan Ge

**Affiliations:** ^1^ Department of Obstetrics and Gynecology, The Second Affiliated Hospital and Yuying Children’s Hospital, Wenzhou Medical University, Wenzhou, China; ^2^ Department of Anesthesiology, The Second Affiliated Hospital and Yuying Children’s Hospital, Wenzhou Medical University, Wenzhou, China; ^3^ Department of Andrology, Chengdu Xi’nan Gynecological Hospital, Sichuan, China; ^4^ Department of Cell Biology & Guangdong Provincial Key Laboratory of Bioengineering Medicine, Jinan University, Guangzhou, China

**Keywords:** luteinizing hormone, testosterone, development, immature Leydig cell, regulation, steroidogenesis

## Abstract

The function of immature Leydig cells is regulated by hormones, such as androgen and luteinizing hormone (LH). However, the regulation of this process is still unclear. The objective of this study was to determine whether luteinizing hormone (LH) or androgens contribute to this process. Immature Leydig cells were purified from 35-day-old male Sprague Dawley rats and cultured with LH (1 ng/ml) or androgen (7α-methyl-19- nortestosterone, MENT, 100 nM) for 2 days. LH or MENT treatment significantly increased the androgens produced by immature Leydig cells in rats. Microarray and qPCR and enzymatic tests showed that LH up-regulated the expression of *Scarb1*, *Cyp11a1*, *Cyp17a1*, and *Srd5a1* while down-regulated the expression of *Sult2a1* and *Akr1c14*. On the contrary, the expression of *Cyp17a1* was up-regulated by MENT. LH and MENT regulate Leydig cell function through different sets of transcription factors. We conclude that LH and androgens participate in the regulation of rat immature Leydig cell function through different transcriptional pathways.

## Introduction

Testosterone secreted by adult Leydig cells in the testis is the main androgen in the male circulation. In mature men, testosterone plays a key role in regulating spermatogenesis and maintaining secondary sexual characteristics (such as increased bone mass, muscle strength, and growth of body hair) (1).

Adult Leydig cells are developed from stem cells during puberty (1, 2). In the rat model, the postnatal development of adult Leydig cells can be conceptually divided into four different stages: stem cells, progenitor cells, immature cells, and adult Leydig cells (1, 3). Stem Leydig cells are the spindle-shaped cells that can differentiate into the Leydig cell lineage (4–6). The first committed cell in this lineage is the progenitor Leydig cell (1). It is also spindle-shaped and expresses the Leydig cell lineage biomarker cytochrome P450 cholesterol side chain cleavage enzyme (CYP11A1, encoded by *Cyp11a1*), 3β-hydroxysteroid dehydrogenase 1 (HSD3B1, encoded by *Hsd3b1*), and cytochrome P450 17α-hydroxylase/17,20-lyase (CYP17A1, encoded by *Cyp17a1*) and appears in interstitial compartment of the testes about 11 to 14 days after birth (1, 3). Progenitor Leydig cells do not have the last step of androgen synthetic enzyme 17β-hydroxysteroid dehydrogenase 3 (HSD17B3, encoded by *Hsd17b3*), which can catalyze the conversion of androstenedione to testosterone (7), but have higher androgen-metabolizing enzymes, including steroid 5α-reductase isoform 1 (SRD5A1, encoded *Srd5a1*) and 3α-hydroxysteroid dehydrogenase (AKR1C14, encoded by *Akr1c14*), which metabolize androstenedione to androstanedione, and further into androsterone (7). In the first week after the appearance of progenitor Leydig cells, they rapidly divide to increase the number of Leydig cells (8), and then differentiate into oval immature Leydig cells around 28 days postpartum (9, 10). Immature Leydig cells express the last-step androgen synthetic enzyme HSD17B3 that catalyzes androstenedione into testosterone, which is further converted into 5α-androstane-3α,17β-diol (androstanediol, DIOL), because these cells still have high capacity of androgen-metabolic enzymes SRD5A1 and AKR1C14 (7). The development of adult Leydig cells from progenitor cells includes an increase of androgen synthetic enzymes (CYP11A1, HSD3B1, CYP17A1, and HSD17B3) and a decrease of androgen metabolic enzymes (SRD5A1 and AKR1C14) (7).

In immature Leydig cells, which hormone controls the proliferation and differentiation of Leydig cells, it is still unclear, but many factors are involved. These factors include luteinizing hormone (LH), insulin-like growth factor-1, transforming growth factor α, transforming growth factor β, interleukin 1, thyroid hormone, anti-Müllerian hormone, and androgen (1). LH has been shown to be an important regulator of Leydig cell function (5, 11). LH works by binding to the LH receptor (LHCGR, encoded by *Lhcgr*) on the Leydig cell membrane, activating adenyyl cyclase to increase intracellular cAMP levels, thereby activating protein kinase A transcription factors (CREB and CREM) signals to regulate Leydig cell function (12). Indeed, knocking out *Lhcgr* in mice resulted in functional defects in Leydig cells, and there are almost no mature adult Leydig cells, only some progenitor Leydig cells exist (13, 14). Besides LH, other factors have yet to be determined. Rat immature Leydig cells contain the highest level of androgen receptor (NR3C4) (9). The role of NR3C4 in the development and function of Leydig cells is more complex, depending on the maturity of Leydig cells. Studies using feminized testis mice (Tfm) with *Nr3c4* null mutations have demonstrated the role of NR3C4 in Leydig cells (15). Despite the high circulating levels of LH, the Tfm had a significant reduction in testosterone production (15–17). In fact, the activity of CYP17A1 and HSD17B3 enzymes is significantly reduced in Tfm testes (15–17). Conditional knockout of *Nr3c4* in mouse Leydig cells also demonstrated that autocrine NR3C4 signal is necessary for the maturation of adult Leydig cells and the regulation of steroidogenic enzymes (18). Although there is a lot of evidence that NR3C4 is important in the early stages of Leydig cell function, such as the progenitor Leydig cell stage (19, 20), NR3C4 signaling in the maturation of immature Leydig cells to adult Leydig cells has not been determined and the detailed signaling for NR3C4 is unclear. The objective of this study was to investigate and compare the effects of LH and androgen signals on the function of rat immature Leydig cells.

## Materials and Methods

### Materials

Ovine LH was a gift from the National Institute of Diabetes and Digestive and Kidney (USA). Since the immature Leydig cells in the 35-day-old rat testis have higher SRD5A1 and AKR1C14 (7), they easily metabolize testosterone to the weak androgen androstanediol, so a synthetic SRD5A1-resistant androgen 7α-methyl-19-nortestosterone (MENT) was used (7, 21). MENT is more potent than testosterone in specifically binding NR3C4 (7, 21). MENT was obtained from Upjohn (Kalamazoo, MI). Sprague-Dawley rats were purchased from Shanghai Laboratory Animal Center (Shanghai, China). The animal experiment protocol was approved by the Institutional Animal Care and Use Committee of Wenzhou Medical University and carried out in accordance with the National Institutes of Health Guide for the Care and Use of Laboratory Animals.

### Immature Leydig Cell Isolation

The method of isolating immature Leydig cells was previously described (19). Briefly, testes from eighteen 35-day-old rats were taken out to isolate immature Leydig cells. The decapsulated testes were dispersed in M199 medium with 0.25 mg/ml collagenase D (Sigma, St. Louis, MO) at 34°C with shaking (75 rpm) for 10 min. The separated cells were filtered through two layers of 100-μm nylon mesh, centrifuged at 250*g*, and resuspended in 55% isotonic Percoll. Subsequently, a density gradient centrifugation was performed in Percoll at 25,000*g* at 4°C for 45 min. The density of the immature Leydig cell fraction collected was between 1.070 and 1.080 g/ml. The cells were washed with Hank’s buffered saline, centrifuged at 250*g*, and then resuspended in phenol red-free Dulbecco’s modified Eagle medium:Nutrient Mixture F-12 (DMEM-F12) medium (Sigma, St. Louis, MO) supplemented with 1 mg/ml bovine serum albumin. According to the published method (22), the purity of immature Leydig cells was determined by histochemical staining of HSD3B1 using 0.4 mM etiocholanolone as a substrate and NAD^+^ as a cofactor. The enrichment of immature Leydig cells was typically as high as 95%. In total, eight isolations were performed.

### Immature Leydig Cell Culture

To test the effects of MENT and LH on androgen synthesis and metabolism, we isolated immature Leydig cells. 1 × 10^6^ Leydig cells were seeded in a 12-well plate and cultured with different concentrations of MENT (10, 100, and 500 nM) or LH (0.1, 1, and 5 ng/ml) or MENT (100 nM) + LH (1 ng/ml) for 48 h. Testosterone and androstanediol from the culture medium were measured and then compared with the control (untreated, basal). Immature Leydig cells were washed with phosphate-buffered saline (PBS) twice and warmed 1× trypsin–EDTA solution (ThermoFisher Scientific Waltham, MA) was added, and the cells were harvested. An aliquot of cell solution was added to a hemocytometer for counting Leydig cell number. Cell viability was checked by the exclusion of 0.4% trypan blue using a kit (ThermoFisher Scientific Waltham, MA) as previously described (23).

### RNA Extraction

According to the manufacturer’s instructions (Invitrogen, Carlsbad, CA), total RNA was extracted from Leydig cells by homogenization in TRIzol. Briefly, rat immature Leydig cells were harvested and homogenized in TRIzol reagent and then extracted with chloroform. The aqueous supernatant containing RNA was retained and the RNA was precipitated with isopropanol. The RNA pellet was eluted with 70% ethanol, air dried, and resuspended in RNase-free water. The RNA was further purified using the RNeasy Kit according to the manufacturer’s instructions (Qiagen, Valencia, CA). A NanoDrop 2000 spectrophotometer (Fisher Scientific, NJ) was used to determine the RNA concentration, and an Agilent 2100 bioanalyzer (Santa Clara, CA) was used to determine the integrity in each individual RNA sample.

### Microarray Hybridization and Scanning

Three independently isolated batches of immature Leydig cells treated with LH and/or MENT in each group were used for the microarray array. The whole genome-expressed RatRef-12 Expression BeadChip from Illumina Inc (San Diego, CA) was used. Each BeadChip contains 21,910 genes, which are mainly selected from the NCBI RefSeq database covering the entire rat transcriptome. As previously reported (24), probe labeling, hybridization, washing, and scanning were performed according to the manufacturer’s instructions using the Illumina Total Prep kit (Applied Biosystems, Foster City, CA). First strand cDNA was synthesized in a total volume of 20 μl with the supplied reagents. The complete first-strand product was used for second-strand synthesis and then subjected to column purification. The purified product was then used for *in vitro* transcription using T7 polymerase. Biotin-16-dUTP was incorporated in this step, resulting in a biotinylated complementary RNA (cRNA) probe. An Agilent 2100 bioanalyzer was used to verify the integrity of the probe. The labeled cRNA (750 ng) was hybridized with the array overnight at 58°C in a total volume of 30 μl of hybridization buffer, followed by rigorous washing and scanning after hybridization.

### Microarray Data Analysis

Scanned microarray expression data was imported into BeadStudio (Illumina, San Diego, CA) for normalization, preliminary analysis, and filtering. Average normalization without background subtraction was used, and the Illumina custom error model was used to generate present/absent calls for each probe (“present” defined as *p* < 0.01 for signal detection) on each array and to call differentially expressed genes at each of samples (defined as *p* < 0.05 after false discovery rate correction). Normalized data from BeadStudio was filtered to exclude genes that were not expressed in the testis (i.e., data from probes that were classed as “absent” in all samples). The net count of target mRNA was calculated by subtracting the background count from the mRNA count. Of the 21,910 genes present in the data, further analysis was conducted. In BeadStudio, linear plot comparison between groups was made, and Heatmap profiling was generated. The data were further imported into Microsoft Access 2010, and queries were generated to find Leydig cell specific genes.

### Biological Pathway Analysis

In order to characterize the biological process, Gene MicroArray Pathway Profiler 2.1 (GenMAPP2.1, San Francisco, CA) software was used to generate a list of significant (P < 0.05) regulatory pathways, and GenMAPP2.1 was used to create a map of signaling pathways. We imported the statistical results into the program and illustrated biological pathways containing differentially expressed genes. The results of the differential gene expression profile were validated by qPCR.

### Quantitative Real-Time PCR

After reverse transcription of the isolated RNA, the level of specific mRNA species was measured by quantitative real-time PCR (qPCR) using the SYBR method. Briefly, first strand synthesis and qPCR were performed as previously described (25). QPCR was conducted in a volume of 20 µl using a 96-well plate format by SYBR Green PCR Core Reagents (Applied Biosystems, Foster City, CA). Primer titration was performed at a concentration of 300 nM. ABI 7700 system (PE Applied Biosystems) was used to detect fluorescence. Each sample was run in duplicate and in parallel with no template controls. The relative mRNA level of the targeted gene was normalized to ribosomal protein S16 (*Rps16*, the house-keeping gene as an internal control) by standard curve method. Ct was read, a standard curve was generated, and the RNA expression in each sample was calculated. All primers in this study were designed by Primer 3 software (Whitehead Institute for Biomedical Research, Cambridge, MA). Forward and reverse primers were placed in different exons to minimize the effects of possible DNA contamination. These genes measured in this study are: luteinizing hormone receptor (*Lhcgr*), scavenger receptor class B member 1 (*Scarb1*), steroidogenic acute regulatory protein (*Star*), *Cyp11a1*, *Hsd3b1*, *Cyp17a1*, *Hsd17b3*, *Srd5a1*, *Akr1c14*, *Insl3*, and *Ccnd1*. We also examined the biomarkers of mature Leydig cells: *Cdk1na*, *Svs5*, and *Ptgds*. Some primers were used in these papers (25, 26) and were listed in the **Supplementary Table S1**.

### Western Blotting Analysis

Cells were homogenized and boiled in an equal volume of sample loading buffer, a Tris-Cl buffer (pH 6.8), containing 20% glycerol, 5% sodium dodecyl sulfate, 3.1% dithiothreitol, and 0.001% bromophenol blue. A homogenized sample (50 µg protein) was electrophoresed on a 10% polyacrylamide gel containing sodium dodecyl sulfate. Proteins were transferred onto a nitrocellulose membrane, and after 1-h exposure to 5% skim milk to block nonspecific binding, the membrane was incubated with CYP17A1 (Abcam Trading Company Ltd. Shanghai, China; dilution, 1:1,000) and actin β (ACTB, Cell signaling technology, Danvers, MA; dilution, 1:1,000) antibody. The membrane was then washed and incubated with a goat anti-rabbit antiserum conjugated to horseradish peroxidase diluted 1:5,000. The washing steps were repeated, and the immunoreactive bands were visualized by chemiluminescence using Amersham ECL kit (Arlington Heights, IL). The density was calculated by ImageJ software (National Institutes of Health, USA) and the density of CYP17A1 relative to ACTB was calculated.

### Medium Testosterone and Androstanediol Measurement

Sephadex LH-20 (Pharmacia Biotech, Uppsala, Sweden) column chromatography was used to separate testosterone and androstanediol as previously described (7). The elution system is chloroform: butane: ethanol (50:50:1, v/v/v) saturated with distilled water. Clear separation of testosterone and androstanediol in this system was confirmed using radiolabeled steroids (7). Separated testosterone and androstanediol were collected and their amounts were measured with a tritium-based RIA as previously described (27). In brief, samples were incubated with a mixture of rabbit testosterone antiserum and ^3^H-testosterone (for testosterone assay) or a mixture of rabbit DIOL antiserum and ^3^H-androstanediol (for androstanediol assay) at 4°C overnight. Testosterone or androstanediol standards (10–2,000 pg/100 µl) were measured in parallel. Bound steroid was separated from the free steroid by mixing with dextran-coated activated charcoal followed by centrifugation. The bound supernatant was put in a bottle with a scintillation cocktail and the relative amount of radioactivity was counted in β-counter (Packard, Meriden, CT). The coefficient of inter-assay and intraassay was 7–8%.

### Analysis of Steroidogenic Enzyme Activities

CYP11A1, HSD3B1, CYP17A1, HSD17B3, SRD5A1, and AKR1C14 activities of intact immature Leydig cells isolated *in vitro* were measured according to the method previously described (7). In brief, an unlabeled steroid (final concentration being 20 μM) spiked with its respective radiolabeled substrate (1 m Ci) for CYP11A1 (^3^H-hydroxychloesterol), HSD3B1 (^3^H-pregnenolone), CYP17A1 (^3^H-progesterone), and HSD17B3 (^3^H-androstenedione), SRD5A1 (^3^H-testosterone), and AKR1C14 (^3^H-dihydrotestosterone) was added. The reaction was started by adding an aliquot of 0.1 × 10^6^ Leydig cells to the reaction mixture. Since testosterone can be metabolized by 5α-reduction in immature Leydig cells, 4-methyl-aza-3-oxo-5 -pregnan-20(S)-carboxylate (2 μM) was used to inhibit SRD5A1 when the activity of CYP17A1 and HSD17B3 measured. The reaction was maintained at pH 7.2. The reaction mixtures, conducted in triplicate, were maintained at 34°C in a shaking water bath (75 rpm) for 10 min. The radioactive products were produced. Reactions were terminated by adding ice-cold ether, and steroids were rapidly extracted. The ether layer was dried under nitrogen. The extracted steroid was dissolved in 100 μl ether and plotted on a thin-layer chromatography plate. The activity of HSD3B1 was determined by measuring the conversion of pregnenolone to progesterone. The activity of CYP17A1 was determined by measuring the conversion of progesterone to 17α-hydroxyprogesterone and androstenedione. The activity of HSD17B3 was determined by measuring the conversion of androstenedione to testosterone. The activity of SRD5A1 was determined by measuring the conversion of testosterone to dihydrotestosterone. The activity of AKR1C14 was determined by measuring the conversion of dihydrotestosterone to androstanediol. Steroids were separated on the thin layer chromatography plate in chloroform-methanol (97:3, v/v) for HSD3B1, HSD17B3, and SRD5A1 assays, in diethyl ether-acetone (98:2, v/v) for AKR1C14, as well as chloroform-ether (7:1, v/v) for CYP17A1 assay. Radioactivity was measured by System 200/AC3000 radioactive scanner (Bioscan, Washington DC).

The activity of CYP11A1 was determined by measuring the conversion of side-chain labeled ^3^H-hydroxycholesterol to radioactive 4-hydroxyl-4-methyl-pentanoic acid as previously described (7). In brief, incubation was performed at 34°C for 30 min, and after incubation, 0.5 ml NaOH (0.5 M) was added. The mixture was extracted twice with 2 ml of chloroform and mixed with neutral alumna to remove non-metabolized substrate, and an aliquot was removed for measurement by liquid scintillation counting on a scintillation counter (Packard, Meriden, CT).

### Statistics

The data were analyzed by Student’s t-test to find significant differences when comparing the two groups (if there were two treatments). The data were analyzed by ANOVA, and then post doc by Tukey’s multiple comparison test to determine significant differences between both groups when comparing three or more groups (including microarray and qPCR data). All data are expressed as means ± SE. The difference is considered significant at *p* < 0.05.

## Results

### Effects of MENT and LH on Androgen Production in Immature Leydig Cells

**Figure 1:**
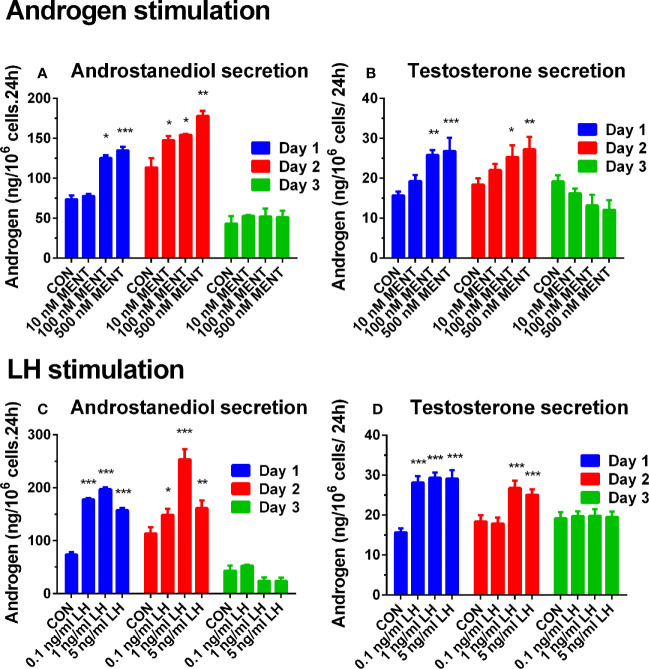
Effect of hormone treatment on androgen production in rat immature Leydig cells. Immature rat Leydig cells were cultured with MENT or LH for 3 days. The levels of medium testosterone and androstanediol were measured. **(A**, **B)** MENT treatment. **(C**, **D)** LH treatment. **(A**, **C)** Androstanediol level. **(B**, **D)** Testosterone level. Mean ± SEM, n = 6. *, **, and *** represent significant differences compared with the control group each day at P < 0.05, 0.01, and 0.001, respectively.

First, we established culture conditions for immature Leydig cells treated with MENT and LH. After 2 days of culture, LH and MENT did not alter the number of immature Leydig cells and cell viability. On days 1 and 2 after culture, immature Leydig cells can maintain the production of androstanediol and testosterone. However, compared with the value on day 1 (73.600 ± 4.77 ng/10^6^ cells.24 h, Mean ± SD, n = 6), androstanediol production of immature Leydig cells in the control was significantly reduced on day 3 (43.395 ± 9.41 ng/10^6^ cells 24 h), while maintaining testosterone production. As shown in **Figure 1A** (androstanediol) and **Figure 1B** (testosterone), MENT showed a stimulating effect on androgen production on days 1 and 2 at 100–500 nM. On the third day, MENT lost its stimulation. As shown in **Figure 1C** (androstanediol) and **Figure 1D** (testosterone), the stimulating effect of LH on androgens on days 1 and 2 was 0.1–5 ng/ml. On the third day, LH lost stimulation. LH (1 ng/ml) showed the highest increase in androstanediol and/or testosterone production on day 2.

**Figure 2:**
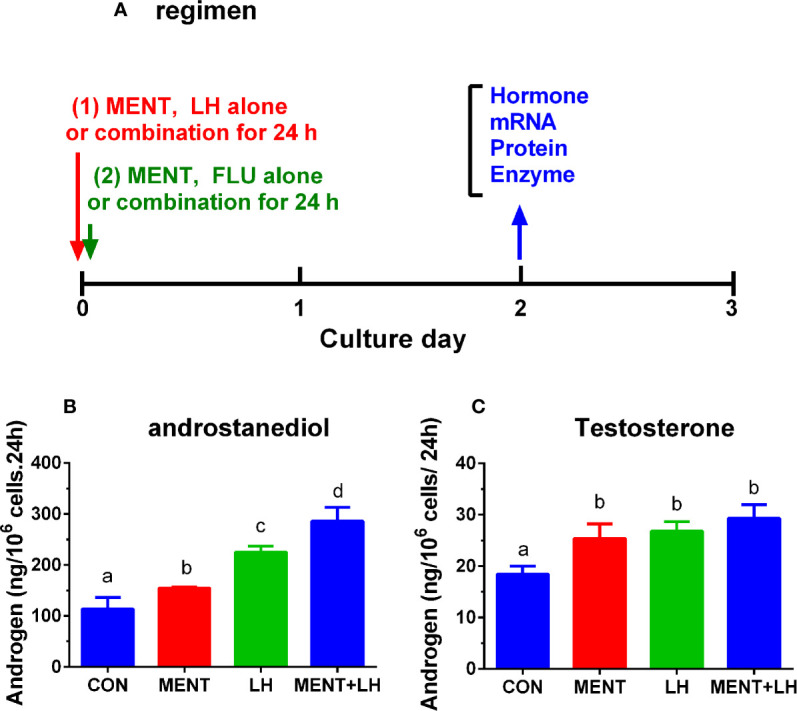
Regimen of treatment and effect of MENT and LH alone or in combination on androgen production in immature Leydig cells in rats. Immature rat Leydig cells were cultured with MENT or LH alone or in combination for 2 days or with FLU or MENT or in combination for another study **(A)**. The levels of medium androstanediol and testosterone were measured. **(A)** Regimen for this experiment and for experiment in **Figures 7** and **8**. **(B)** Androstanediol. **(C)** Testosterone level. Mean ± SEM, n = 4. The same letter means that there is no significant difference between the two groups at P < 0.05.

Then, we used MENT (100 nM) and LH (1 ng/ml) alone or in combination to treat rat immature Leydig cells for 2 days as described in **Figure 2A**. As shown in **Figure 2**, the combination of LH and MENT further increased the levels of androstanediol (**Figure 2A**) without affecting testosterone (**Figure 2B**), indicating that LH and MENT synergistically act on androstanediol production in rat immature Leydig cells.

### Gene Profile of Rat Immature Leydig Cells After LH and MENT Treatment

**Figure 3:**
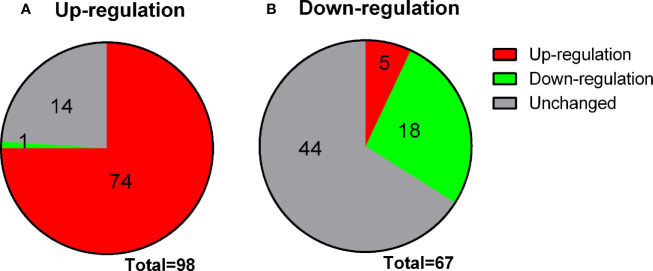
Changes in genes overlap between LH and MENT. **(A)** 98 genes up-regulated by MENT ≥3 times, 74 genes up-regulated by LH ≥3 times, 1 gene down-regulated by LH and 14 unaltered genes; **(B)** 67 genes down-regulated by MENT were 18 genes down-regulated, 4 genes up-regulated and 44 unaltered genes after LH treatment.

Rat immature Leydig cells (three different replicates) were treated for 2 days without treatment (control) or 100 nM MENT, 1 ng/ml LH, or 100 nM MENT + 1 ng/ml LH (MENT + LH). The Illumina RatRef-12 expression chip was used to determine changes in mRNA levels. The whole genome expression containing 21,910 probes was analyzed. The counts of house-keeping gene *Rps16* were 150.00 ± 4.00, 152.97 ± 9.37, 160.94 ± 8.88, and 145.56 ± 8.39, for control, MENT, LH, and MENT + LH, respectively and there was no significant difference between two groups. There was no statistical difference between two groups for *Actb*, another housing gene (data not shown). Among these probes, 8,047 probes were detected in the control group. When compared with the control group, MENT up-regulated 98 genes by approximately three times (**Table 1** lists the genes whose expression has increased five times). Of these genes, 74 were also up-regulated by two folds and higher by LH, and only one (*Aoc3*) was down-regulated by LH (**Figure 3A**). Compared with controls, MENT down-regulated the expression of 67 genes by approximately three times (**Table 2** lists the genes whose expression has decreased five times). Of these 67 genes, 18 genes were also down-regulated ≥2 folds, and only five were up-regulated by LH (**Figure 3B**). This suggests that the up-regulated genes between MENT and LH are mostly overlapping while the down-regulated genes are very different.

**Table 1:** Genes were up-regulated 5 folds or more by androgen (MENT).

Ontogeny	Gene symbol	Arbitrary count			
		Control	MENT	LH	LH+MENT
**Cell cycle**	Cdkn1a	20	160(+8)	161(+8)	178(+8)
**Cytokine**	Cxcl2	119	1250(+10)	5927(+50)	1157(+10)
	Il6	75	644(+9)	1560(+21)	415(+6)
**Extracellular matrix**	Serpine1	1012	4982(+5)	8862(+9)	5070(+5)
	Cyr61	536	2581(+5)	5088(+10)	11970(+4)
	Tfpi2	123	669(+5)	1706(+14)	539(+4)
	Timp3	63	319(+5)	133(+2)	211(+3)
**Growth arrest**	Errfi1	688	6335(+9)	3362(+5)	2667(+4)
	Gadd45g	122	2071(+17)	488(+4)	689(+6)
**GTP binding**	Arl5b	56	431(+8)	251(+4)	262(+5)
**Metabolism**	Ptgs2	2160	14724(+7)	25443(+12)	10581(+5)
	Cyp26b1	513	2680(+5)	1522(+3)	1618(+3)
	Aldh5a1	116	653(+6)	193(+2)	401(+3)
	Ero1l	162	876(+5)	583(+4)	491(+3)
**Protein binding**	Cldn1	73	405(+5)	158(+2)	166(+2)
**Growth factor**	Fgl2	247	1653(+7)	297(+1.2)	523(+3)
	Adm	281	3202(+11)	2730(+10)	2025(+7)
	Ctgf	45	521(+12)	1237(+27)	232(+5)
	Adora2a	765	4103(+5)	4143(+5)	3132(+4)
	Gdf15	180	925(+5)	995(+5)	830(+5)
**Signaling**	Csf3	34	181(+5)	1306(+39)	273(+8)
	Pim1	31	355(+11)	166(+5)	196(+6)
	Rgs4	43	418(+10)	674(+16)	291(+7)
	Ppp1r3b	61	427(+7)	232(+4)	396(+6)
**Steroidogenesis**	Cyp17a1	288	1082(+5)	600(+2)	574(+2)
**Transcription factor**	Bhlhb3	217	1781(+8)	1081(+5)	1194(+5)
	Atf3	102	619(+6)	648(+6)	382(+3)
	Hig1	260	1438(+6)	1276(+5)	914(+4)
	Mxd1	16	243(+15)	237(+15)	272(+17)
	Bhlhb2	1248	6539(+5)	3926(+3)	4554(+4)
	Bhlhb3	182	1745(+10)	1044(+6)	1158(+6)
	Btg2	423	2116(+5)	3114(+7)	1552(+4)
**Transporter**	Slc40a1	295	2225(+7)	306(+1)	624(+2)
	Atp2b3	96	597(+5)	76(-0.9)	140(+1.4)
	Slc2a3	26	193(+7)	153(+6)	147(+6)

() Fold changes after MENT, LH or LH+MENT compared to control. 35 Genes examined, of them, the expression of 32 genes were up-regulated by 2 folds or higher by LH treatment.

**Table 2:** Genes were down-regulated 5 folds or more after androgen (MENT).

Ontology	Symbol	Arbitrary count			
		CON	MENT	LH	LH+MENT
**Cytokine**	Cxcl12	3151	427(-7)	477(-7)	390(-8)
	Ier5l	552	50(-11)	150(-4)	253(-2)
	Xcl1	962	111(-9)	554(-2)	174(-6)
	Cxcl11	3126	506(-6)	1715(-2)	368(-9)
	Cxcl13	283	28(-10)	961(+3)	189(-0.5)
**Extracellular matrix**	Slpi	363	14(-26)	872(+2)	223(-2)
	Mmp12	392	67(-6)	880(+2)	91(-4)
**Metabolism**	Ubd	6705	778(-9)	3930(-2)	776(-9)
	Es1	11891	2051(-6)	10641(-1.1)	3524(-3)
**Protein binding**	Sc65	562	91(-6)	176(-3)	221(-3)
**Receptor binding**	Cd74	439	18(-24)	310(-1.3)	10(-46)
	Tmem178	910	133(-7)	1149(+1.2)	513(-2)
	Chi3l1	213	16(-13)	110(-2)	17(-13)
	Asgr1	315	48(-7)	818 (+2)	473(+1.4)
	Tmem15	153	27(-6)	45(-3)	65(-2)
	Ccr5	324	71 (-5)	222(-1.3)	116(-3)
**Signaling**	Igtp	788	164(-5)	384(-2)	246(-3)
	Sh2d2a	208	31(-7)	56(-4)	33(-6)
	Serpina3n	2744	579(-5)	2346(-1.2)	1399(-2)
**Transcription factor**	Snrpa	1021	229(-5)	1533(+1.5)	558(-2)
	Aif1	161	33(-5)	92(-2)	48(-3)
**Transporter**	Pla2g2a	7253	167(-43)	4179(-2)	781(-9)
	Hpx	12486	412(-30)	11281(-1.1)	3621(-3)
	S100a4	1588	415(-5)	1027(-1.5)	462(-3)
	Lcn2	4902	568(-8)	3090(-1.5)	1436(-3)

() Fold changes after MENT, LH or LH+MENT when compared to control (CON). 25 genes examined, of them, 12 genes were also down-regulated 2 folds or higher by LH treatment.

### Analysis of Steroidogenic Pathways and Cell Cycle Pathways

**Figure 4:**
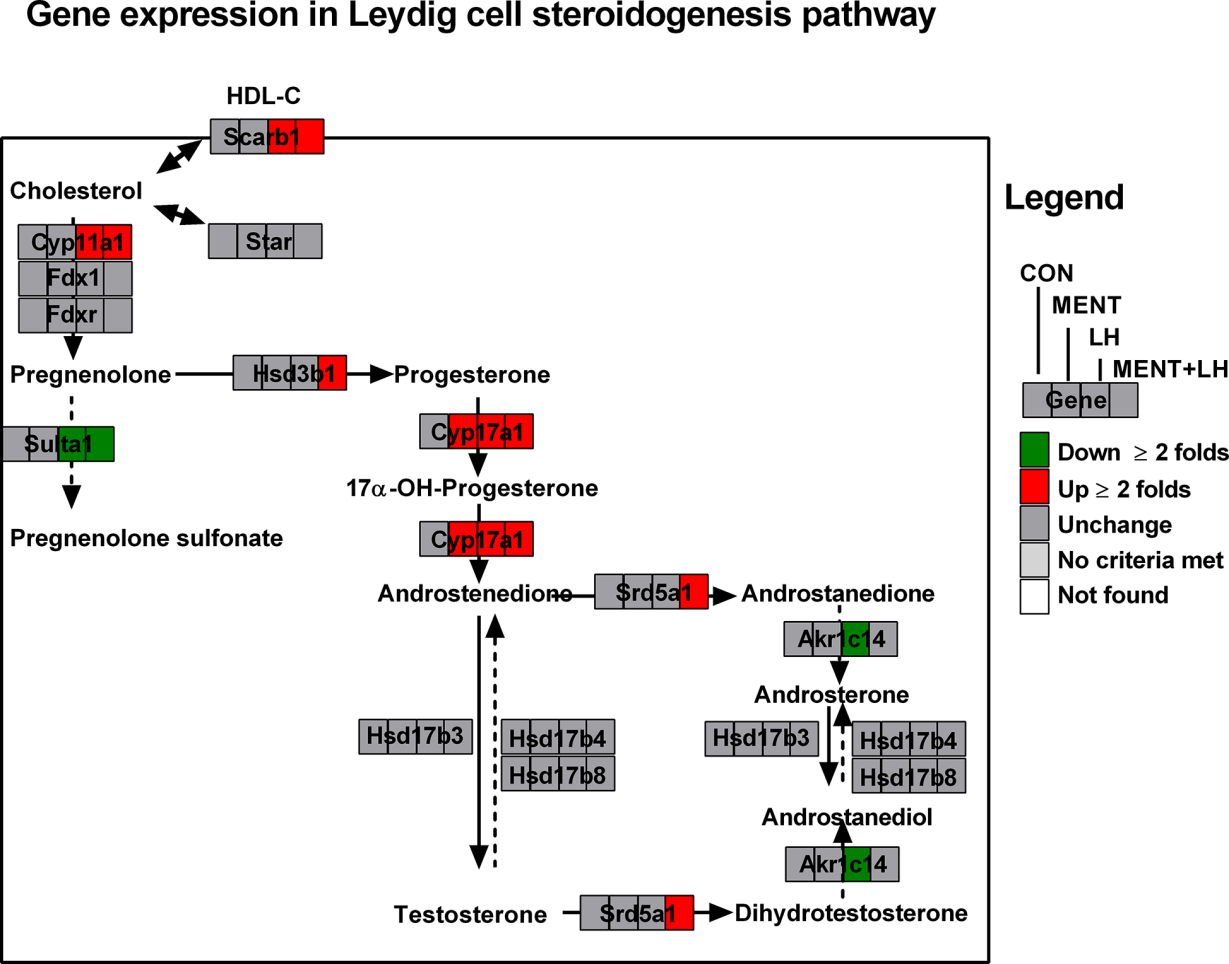
Analysis of gene pathways for MENT, LH alone or in combination (MENT+LH) to regulate steroidogenesis. Red indicates up-regulated genes; green indicates down-regulated genes. Solid arrow indicates androgen synthesis pathway; Dash arrow indicates steroid metabolism; Double arrows indicates transport of cholesterol.

Using GenMAPP2, we discovered several pathways specific to MENT or LH regulation. As shown in **Figure 4** and **Supplementary Table S2**, steroidogenesis-related genes (*Scarb1*, *Cyp11a1*, and *Cyp17a1*) were significantly increased by ≥2 folds, while the two steroid metabolic enzymes (*Sult1a1* and *Akr1c14*) were significantly decreased by ≥2 folds after LH treatment. However, after MENT treatment, only *Cyp17a1* increased significantly by ≥2 folds. This suggests that MENT and LH stimulate the steroidogenesis of immature Leydig cells by targeting different genes. Interestingly, the combination of LH and MENT led to the increases of two additional genes (*Hsd3b1* and *Srd5a1*) besides *Scarb1*, *Cyp11a1*, and *Cyp17a1*, indicating that the combination of LH and MENT is conducive to optimized steroidogenesis.

When we examined cell cycle pathway (**Supplementary Figure S1**), we found that the expression of *Cdkn1a*, *Cdkn1b*, *Gadd45a*, *Ccng2*, and *RGD735212* increased by ≥2 folds after MENT treatment. MENT treatment also resulted in down-regulation of *Ccnd1* and *Pola2*. The expression of *Cdkn1a*, *Gadd45a*, and *Ccng2* was also significantly up-regulated by ≥2 folds after LH treatment. These results indicate that both LH and MENT cause cell cycle arrest by inducing similar cell cycle arrest regulators.

**Figure 5:**
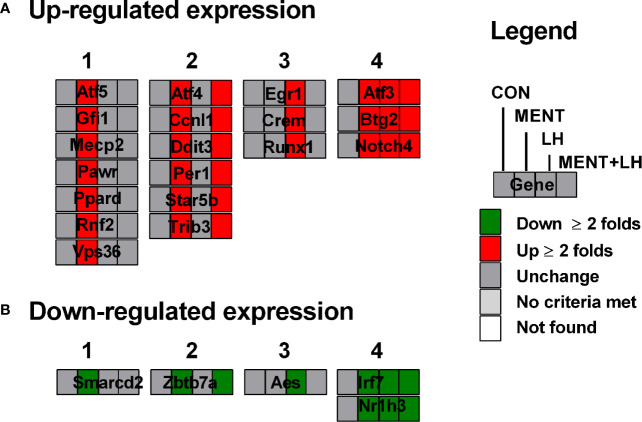
Transcription factors are regulated by MENT, LH alone or in combination (MENT+LH). Red indicates that the gene is up-regulated; green indicates that the gene is down-regulated. **(A)** Up-regulated expression of genes. **(B)** Down-regulated expression of genes; Four categories: (1) Only up-regulated/down-regulated in MENT-treated groups; (2) Up-regulated/down-regulated in MENT alone or MENT+LH groups; (3) Only up-regulated/down-regulated in LH-treated group; (4) Up-regulated/down-regulated in MENT, LH, and MENT + LH groups.

When we examined the transcription pathway, we found that MENT and LH regulated quite different sets of transcription factors. We divided the patterns of gene expression in four categories. As shown in **Supplementary Table S2** and **Figure 5A** (for up-regulated genes), category 1 lists the gene expression only affected by MENT, which contains *Atf5*, *Gfi1*, *Mecp2*, *Pawr*, *Ppard*, *Raf2*, and *Vps36;* category 2 lists the gene expression affected by MENT and MENT+LH, which contains *Atf4*, *Ccnl1*, *Dit3*, *Per1*, *Stat5b*, and *Trib3*); category 3 lists the gene expression only affected by LH, which contains *Egr1*, *Crem1*, and *Runx1;* category 4 lists the gene expression affected by LH, MENT, and MENT + LH, which contains *Atf3*, *Btg2*, and *Notch4.* As shown in **Figure 5B** (for down-regulated genes), category 1 lists the gene expression only affected by MENT, which contains *Smarcd2;* category 2 lists the gene expression affected by MENT and MENT + LH, which contains *Zbtb7a*; category 3 lists the gene expression only affected by LH, which contains *Aes* and *Tceal1;* category 4 lists the gene expression affected by LH, MENT, and MENT + LH, which contains *Irf7* and *Nr1h3.* This suggests that MENT function through divergent transcriptional regulation.

### Comparison of Microarray With qPCR Data

**Figure 6:**
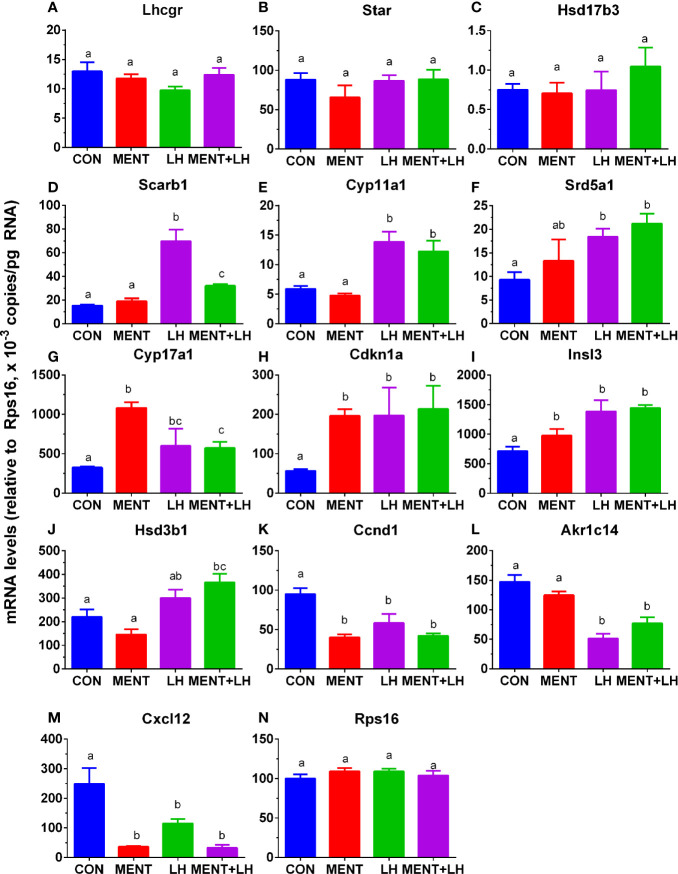
Messenger RNA levels are regulated by MENT or LH alone or in combination (MENT+LH) after qPCR analysis. In the presence of MENT or LH alone or in combination, immature Leydig cells were cultured for 2 days. The following genes were analyzed **(A–N)**: *Lhcgr*, *Star*, *Hsd17b3*, *Scarb1*, *Cyp11a1*, *Srd5a1*, *Cyp17a1*, *Cdkn1a*, *Insl3*, *Hsd3b1*, *Ccnd1*, *Akr1c14*, *Cxcl12*, and *Rps16*. The data are shown as mean ± SEM, n = 4–8 preparations. The same letter indicates that the difference between the two groups is not statistically significant (P < 0.05).

*Rps16* was used as a house-keeping gene. Its levels by qPCR were not significantly changed between groups (control, MENT, LH, and MENT + LH, **Figure 6**). We selected three sets of genes: unchanged after treatment with MENT and LH (*Lhcgr*, *Star*, and *Hsd17b3*), up-regulated by LH (*Scarb1*, *Cyp11a1*, and *Srd5a1*), up-regulated by LH and MENT (*Cyp17a1*, *Cdkn1a*, and *Insl3*), up-regulated only by LH plus MENT (*Hsd3b1*), and down-regulated by LH and MENT (*Ccnd1*, *Akr1c14*, and *Ccx12*) and subjected to qPCR analysis. We found similar trends in these gene changes in the microarray (**Figure 6**), which indicates that the microarray analysis is reliable.

### Identify Whether MENT Action Via Androgen Receptor

**Figure 7:**
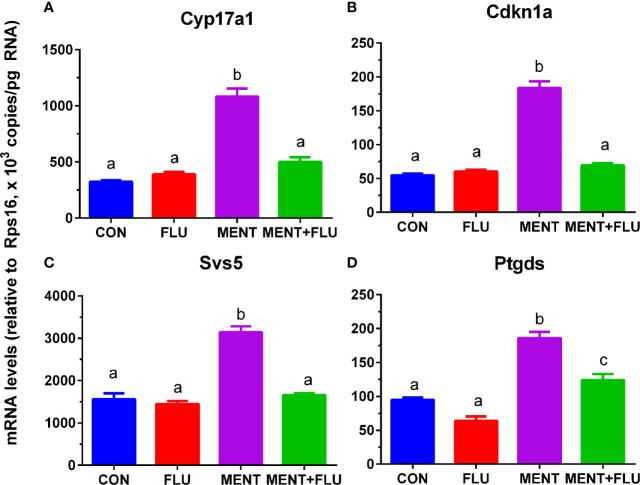
Antagonistic effect of flutamide (FLU) on MENT-induced gene up-regulation. In the presence of MENT, FLU alone or in combination (MENT +FLU), immature Leydig cels were cultured for 2 days. The following genes were analyzed **(A–D)**: *Cyp17a1*, *Cdkn1a, Svs5*, and *Ptgds.* The data are shown as mean ± SEM, n = 4–8 preparations. The same letter indicates that the difference between the two groups is not statistically significant (P < 0.05).

**Figure 8:**
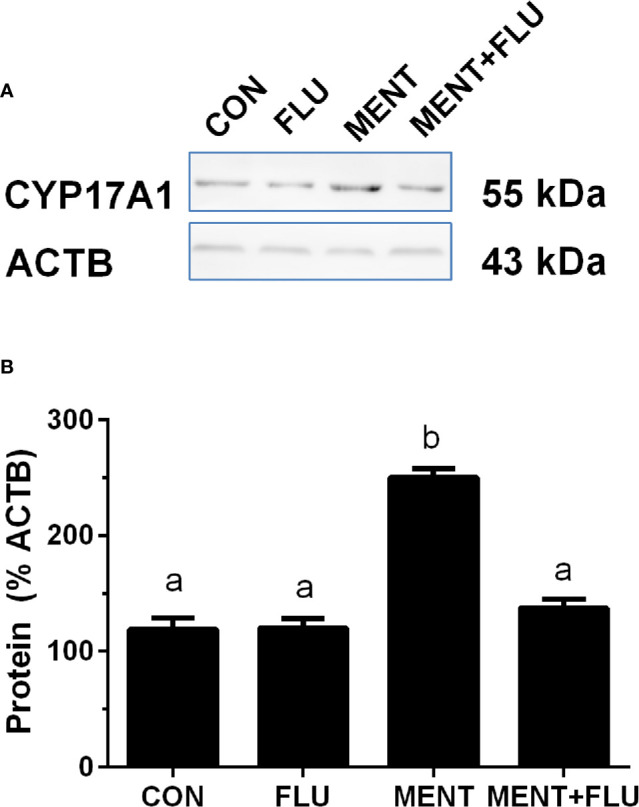
Androgen receptor antagonist flutamide (FLU) antagonizes MENT-induced CYP17A1 level. In the presence of MENT, FLU alone or in combination (MENT +FLU), immature Leydig cells were cultured for 2 days. Analysis of CYP17A1 and actin β (ACTB) levels. The CYP17A1 level was adjusted to ACTB. **(A)** Western blot; **(B)** Quantitative data. The data is shown as mean ± SEM, n = 3 preparations. The same letter indicates that the difference between the two groups is not statistically significant (P < 0.05).

We treated rat immature Leydig cells for 2 days without (control), 1 µM flutamide (FLU), 100 nM MENT, and 1 µM FLU and 100 nM MENT (FLU + MENT), and selected four genes (*Cyp17a1*, *Cdkn1a*, *Svs5*, and *Ptgds*) are biomarkers of mature Leydig cells and can be analyzed by qPCR. We found that androgen receptor antagonist flutamide reversed the regulation of these genes by MENT (**Figure 7**). This suggests that MENT up-regulates these genes though androgen receptors. We chose a protein CYP17A1 and found that the level of CYP17A1 changed with its mRNA (**Figure 8**).

### Identification of Enzyme Activity After LH or MENT Treatment

**Figure 9:**
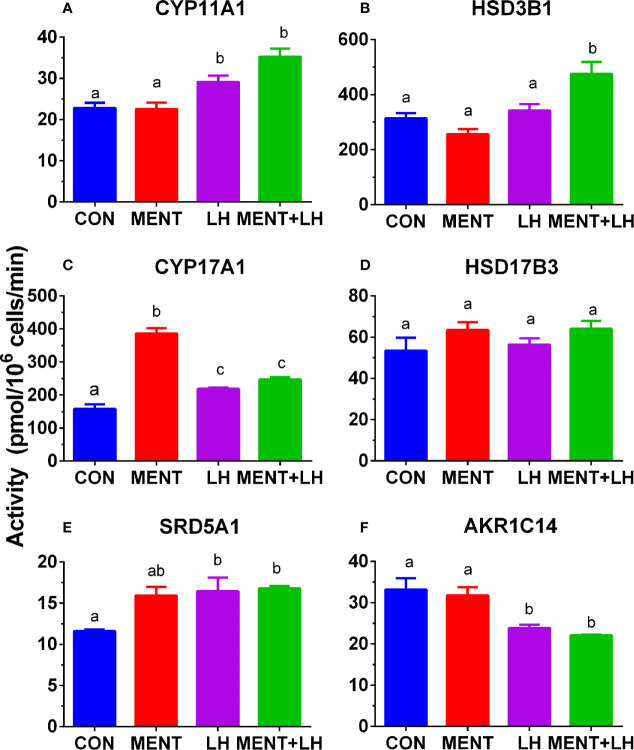
Effect of MENT, LH alone or in combination (MENT+LH) on the activity of steroidogenic enzymes in immature Leydig cells. In the presence of MENT, LH alone or in combination (MENT+LH), immature Leydig cells were cultured for 2 days. **(A–F)**: CYP11A1, HSD3B1, CYP17A1, HSD17B3, SRD5A1, AKR1C14, respectively. The data is shown as mean ± SEM, n = 4 preparations. The same letter indicates that the difference between the two groups is not statistically significant (P < 0.05).

We performed enzyme activity assays to identify protein changes in CYP11A1, HSD3B1, CYP17A1, HSD17B3, SRD5A1, and AKR1C14 in rat immature Leydig cells using radioactive substrates. As shown in **Figure 9**, the activity of all enzymes paralleled changes in their mRNA levels.

## Discussion

This study shows that androgen (MENT) and LH stimulate the function of immature Leydig cells in rats. androgen (MENT) and LH regulate different sets of transcription factors to control steroidogenesis. MENT mainly binds to NR3C4 to up-regulate the expression of *Cyp17a1*. LH mainly up-regulates the expression of *Cyp11a1*, *Cyp17a1*, and *Srd5a1* through *Crem1* and *Egr1* transcription factors. However, LH down-regulates the expression of steroid metabolic enzyme genes, *Akr1c14* and *Sult1a1*, thereby increasing the androgen formation of rat immature Leydig cells.

LH is essential for Leydig cell function because it is the principle stimulating hormone of androgen production in adult Leydig cells (1). Previous studies have shown that LH stimulated the development of rat progenitor Leydig cells into immature Leydig cells (5, 11, 20). In this study, we used 1 ng/ml LH instead of 100 ng/ml because high concentration of LH can cause LHCGR internalization (28, 29), and we also used a 2-day culture in which immature Leydig cells still maintained strong androgen production. Obviously, LH (1 ng/ml) can increase androgen production by two to three times, which is reasonable because these cells have lower LHCGR number compared to adult Leydig cells (9). In this study, we also demonstrated that LH stimulated the function of immature Leydig cells. In the steroidogenic pathway, LH up-regulated the expression of *Scarb1*, *Cyp11a1*, and *Cyp17a1*, and down-regulated the expression of *Sult1a1* and *Akr1c14* (Fig.4), thereby increasing androgen synthesis. Our previous studies have demonstrated that immature Leydig cells matured into adult Leydig cells by increase in the expression of *Scarb1*, *Cyp11a1*, and *Cyp17a1* and down-regulation of *Akr1c14* (7, 30). Therefore, LH is an important factor for function and differentiation of immature Leydig cells. The combination of MENT and LH also significantly down-regulated the expression of *Akr1c14* (by 1.9 folds, **Supplementary Table S2**). The up-regulation of other biomarker of mature Leydig cells such as *Insl3* also suggests that LH is a factor for inducing the differentiation of immature Leydig cells.

LH has been demonstrated to regulate Leydig cell function by binding to LHCGR, activating adenylyl cyclase to increase cAMP levels, thereby activating PKA/CREB/CREM signals (12). Knocking out *Lhcgr* in mice led to defects in the function of Leydig cells with no adult Leydig cells in the adult testis (13, 14). Indeed, in the current study, we found that CREM was significantly up-regulated (**Figure 5**). Although individual studies identified that several pathways besides cAMP/PKA signal are also involved, including MEK/ERK1/2 signal, the present study provided the transcription profiles of LH-mediated action in immature Leydig cells *via* several other pathways, including LHCGR/EGR1, Runx1 et al. (**Figure 5**).

In the steroidogenic pathway, MENT is different from LH, and only up-regulates the expression of *Cyp17a1*. Earlier evidence from the Tfm model supports the role of NR3C4 signaling in Leydig cell function. The expression levels of Leydig cell maturation markers *Insl3* and *Cyp17a1* in mice lacking functional NR3C4 were significantly reduced (16, 31). Although the role of NR3C4 in other cell types in Tfm cannot be ruled out, Leydig cell condition knockout mice for *Nr3c4* also resulted in a significant reduction in mature Leydig cell biomarkers (*Cyp17a1*, *Hsd17b3*, and *Insl3*) (18). Obviously, NR3C4 acts through the androgen response element of the target gene. In this regard, in the *Insl3* gene, the region that mediates androgen action has been mapped to the sequence of 2132 to 285 in the promoter (32). According to reports (16, 18, 31), in Tfm or Leydig cell conditionally knocked out NR3C4 mice, *Cyp17a1* expression levels were significantly down-regulated. However, there is little information about the NR3C4 stimulation site in *Cyp17a1*. In contrast, in mature Leydig cells, androgens actually inhibit the expression level of *Cyp17a1* through the binding sequence in the cAMP response region of the *Cyp17a1* promoter (33). This difference in the androgen effect on the expression of *Cyp17a1* may depend on the maturity of Leydig cells. Our data also points to androgen stimulation of immature Leydig cell maturation, as indicated by increased expression of *Cyp17a1*, *Cdkn1a*, *Svs5*, and *Ptgds*. *Cyp17a1* (7), *Cdkn1a* (34), *Svs5* (35), and *Ptgds* (36) have been identified as biomarkers for Leydig cell maturation. The increase in the expression of these genes (*Cyp17a1*, *Cdkn1a*, *Svs5*, and *Ptgds*) mediated by MENT was antagonized by flutamide (**Figure 7**), which is an inhibitor of NR3C4, indicating that MENT up-regulates the expression of these genes through androgen receptor.

Interestingly, among the up-regulated genes, MENT and LH showed higher similarity. Of the 98 genes that were up-regulated by MENT three times, 74 were also up-regulated by LH, and only 1 was down-regulated (**Figure 3**). Although some effects of LH on the gene expression may be mediated by testosterone, immature Leydig cells secrete a very low amount of testosterone and the major androgen is androstanediol, which almost has no binding to the androgen receptor. Furthermore, we found that LH and androgen regulate different sets of transcriptional factors related to Leydig cell differentiation. LH significantly up-regulated the expression of *Crem1*, *Atf3*, *Egr1*, and *Runx1* (**Figure 5**), and down-regulated transcription factor *Aes*. The combination of MENT and LH also down-regulated *Aes* expression by 1.9-folds although the significant difference did not reach P < 0.05 (**Supplementary Table S2**). CREM, CREB (cAMP responsive element binding protein), and ATF (activating transcript factor) belong to the CREB transcriptional factor family, they respond to cAMP signaling and bind to cAMP response element (CRE) site in the target gene promoter (37–39). LH-LHCGR-protein kinase A signaling may lead to up-regulation of *Crem1* and *Atf3*. It has been reported that LH up-regulated *Egr1* in gonadal cells (40), which is critical for steroidogenesis in Leydig cells (41). There are also reports that *Runx1* is up-regulated by LH and is involved in steroidogenesis in gonadal cells (42). Thirteen genes (*Atf5*, *Gfi1*, *Mecp2*, *Pawr*, *Ppard*, *Raf2*, *Vps36*, *Atf4*, *Ccnl1*, *Dit3*, *Per1*, *Stat5b*, and *Trib3*) were significantly up-regulated while two genes (*Smarcd2* and *Zbtb7a*) were down-regulated only by MENT by 2-fold (**Figure 5**). These data suggest that LH and MENT function through different transcriptional regulation mechanisms.

In conclusion, this study shows that androgen and MENT can induce immature Leydig cells to differentiate into adult Leydig cells. Androgen and androgen act on their respective receptors and activated different sets of transcriptional factors to regulate the function of immature cells into adult Leydig cells (**Supplementary Figure S2**).

## Data Availability Statement

The raw data supporting the conclusions of this article will be made available by the authors, without undue reservation.

## Ethics Statement

The animal study was reviewed and approved by the Institutional Animal Care and Use Committee of Wenzhou Medical University.

## Author Contributions

XL, QZ, ZW, YW, KY, and ZS performed experiments. KY performed microarray analysis. XL, YZ, and R-SG designed the study. XL drafted the manuscript. R-SG edited the manuscript. All authors contributed to the article and approved the submitted version.

## Funding

The work is supported by the National Natural Science Foundation of China (81730042).

## Conflict of Interest

The authors declare that the research was conducted in the absence of any commercial or financial relationships that could be construed as a potential conflict of interest.

## Supplementary Material

Supplementary Figure 1 Analysis of G1 to S cell cycle-controlled gene pathway regulated by MENT, LH alone or in combination (MENT + LH). Red indicates that the gene is up-regulated; green indicates that the gene is down-regulated.

Supplementary Figure 2 Illustration of androgen (MENT) and luteinizing hormone (LH) regulating steroid synthesis and metabolism in Leydig cells through different pathways and targets. MENT binds to androgen receptor (NR3C4) to up-regulate the expression of *Cyp17a1*. LH binds to its receptor (LHCGR) to trigger the cAMP/PKA pathway, thereby up-regulating the expression of *Scarb1*, *Cyp11a1*, and *Cyp17a1* and down-regulating the expression of *Sult1a1* and *Akr1c14*.

## Abbreviations

MENT, 7α-Methyl-19- nortestosterone; CYP11A1, Cytochrome P450 cholesterol side chain cleavage enzyme; HSD3B1, 3β-Hydroxysteroid dehydrogenase 1; CYP17A1, Cytochrome P450 17α-hydroxylase/17, 20-lyase; HSD17B3, 17β-Hydroxysteroid dehydrogenase 3; SRD5A1, Steroid 5α-reductase isoform 1; AKR1C14, 3α-Hydroxysteroid dehydrogenase; DIOL, 5α-Androstane-3α,17β-diol; LH, Luteinizing hormone; NR3C4, Androgen receptor; Tfm, feminized testis mice.
