# Supplementary figures and images for: Androgen and Luteinizing Hormone Stimulate the Function of Rat Immature Leydig Cells Through Different Transcription Signals
Source: Front Endocrinol (Lausanne). 2021 Mar 17;12:599149. doi: 10.3389/fendo.2021.599149 (PMC8011569; doi:10.3389/fendo.2021.599149)

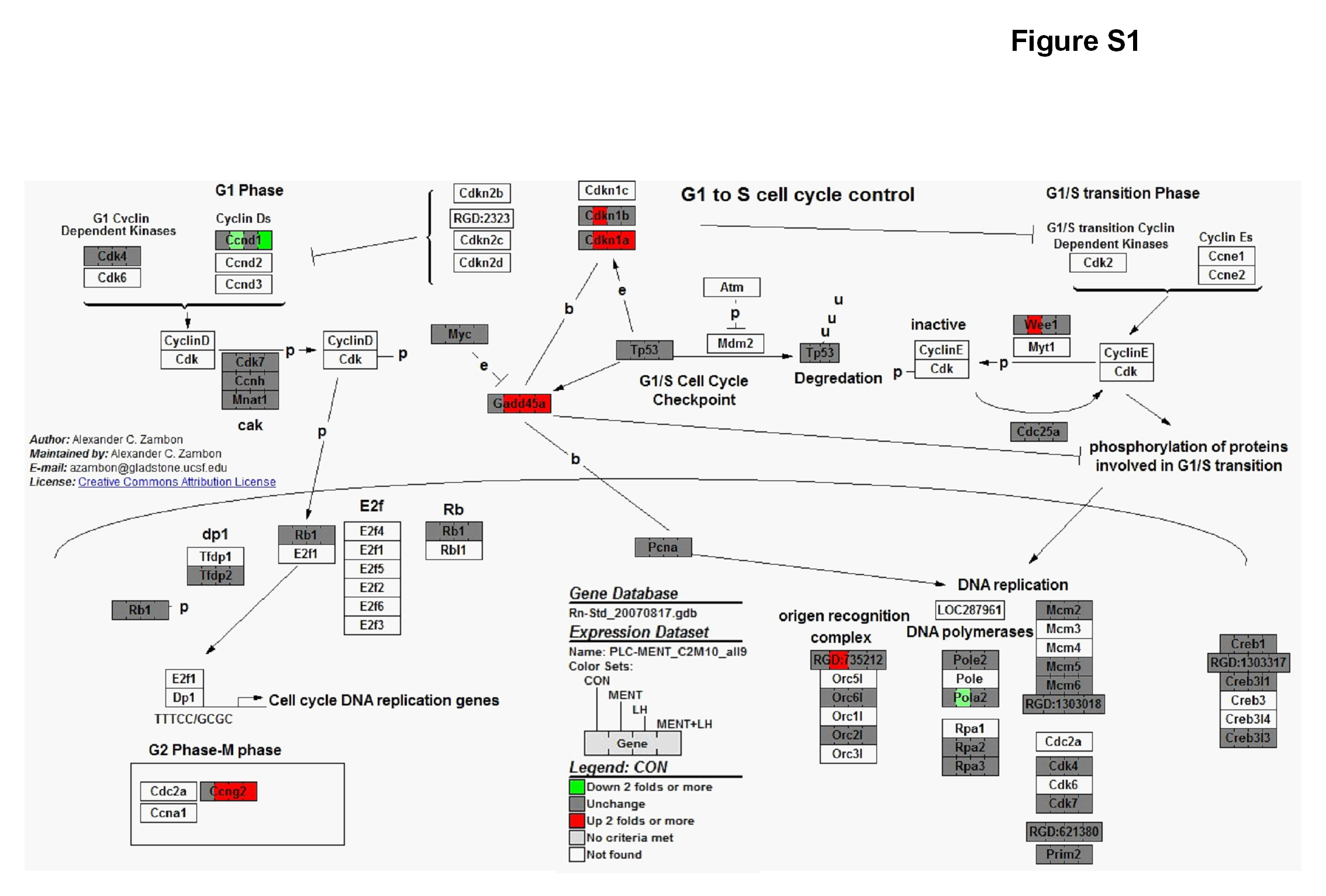

Supplement: Supplementary Figure 1 — Analysis of G1 to S cell cycle-controlled gene pathway regulated by MENT, LH alone or in combination (MENT + LH). Red indicates that the gene is up-regulated; green indicates that the gene is down-regulated. [file Image_1.tif]

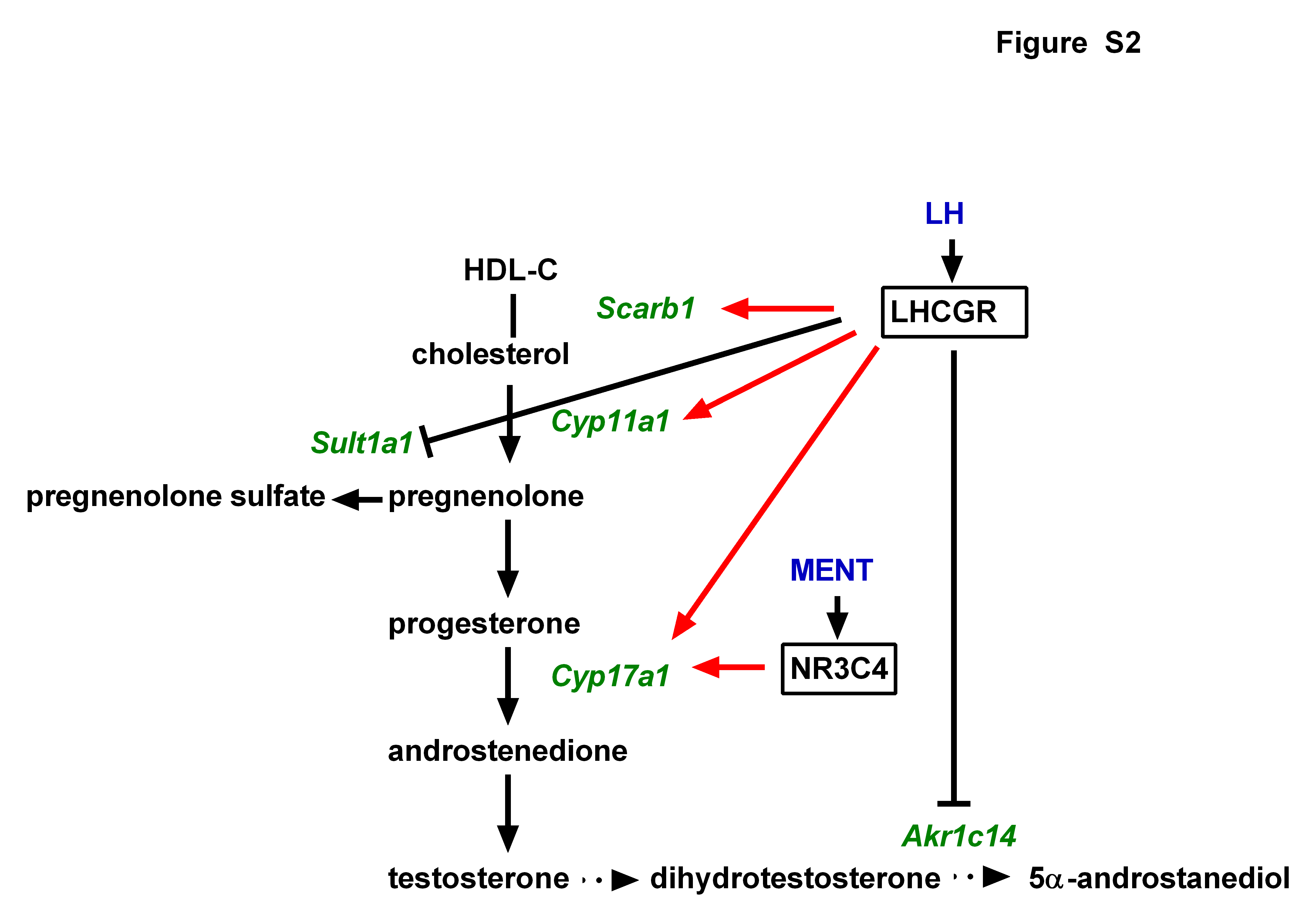

Supplement: Supplementary Figure 2 — Illustration of androgen (MENT) and luteinizing hormone (LH) regulating steroid synthesis and metabolism in Leydig cells through different pathways and targets. MENT binds to androgen receptor (NR3C4) to up-regulate the expression of Cyp17a1. LH binds to its receptor (LHCGR) to trigger the cAMP/PKA pathway, thereby up-regulating the expression of Scarb1, Cyp11a1, and Cyp17a1 and down-regulating the expression of Sult1a1 and Akr1c14. [file Image_2.tif]
