# Supplementary material for: Androgen and Luteinizing Hormone Stimulate the Function of Rat Immature Leydig Cells Through Different Transcription Signals
Source: Front Endocrinol (Lausanne). 2021 Mar 17;12:599149. doi: 10.3389/fendo.2021.599149 (PMC8011569; doi:10.3389/fendo.2021.599149)
Supplement: Supplementary file 3 [file Table_1.doc]

**Supplementary Table S1: Primers for Leydig cells genes (16 genes)**

| **Gene Symbol** | **Accession #** | **Forward Primer** | **Reverse Primer** | **Size (bp)** |
| --- | --- | --- | --- | --- |
| *Insl3* | NM_053680 | 5’GTGGCTGGAGCAACGACA3’ | 5’AGAAGCCTGGTGAGGAAGC3’ | 102 |
| *Lhcgr* | NM_012978 | 5’CTGCGCTGTCCTGGCC3’ Wrong: | 5’CGACCTCATTAAGTCCCCTGAA3’ | 102 |
| *Scarb1* | NM_031541 | 5’ATGGTACTGCCGGGCAGAT3’ | 5’CGAACACCCTTGATTCCTGGTA3' | 117 |
| *Star* | NM_031558 | 5’CCCAAATGTCAAGGAAATCA3’' | 5’AGGCATCTCCCCAAAGTG3’ | 187 |
| *Cyp11a1* | NM_017286 | 5’AAGTATCCGTGATGTGGG3’ | 5’TCATACAGTGTCGCCTTTTCT3’ | 126 |
| *Hsd3b1* | NM_017265 | 5’CCCTGCTCTACTGGCTTGC3’ | 5’TCTGCTTGGCTTCCTCCC3’ | 178 |
| *Cyp17a1* | NM_012753 | 5’TGGCTTTCCTGGTGCACAATC3’ | 5’TGAAAGTTGGTGTTCGGCTGAAG3’ | 90 |
| *Hsd17b3* | NM_054007 | 5’ TTCTTCGGGAGTAGGGGTTC 3’ | 5TCATCGGCGGTCTTGGTCG3’ | 201 |
| *Sdr5a1* | J05035 | 5’TCACCAGAGCGAAGCAGC3’ | 5’CTAAAGCACAAATGGAATCAGTAT3’ | 92 |
| *Akr1c14* | NM_138547 | 5' GCAGCGTGGGGTTGTG3' | 5' TGGATGATTGGGATGGTCA3' | 172 |
| *Ccnd1* | NM_171992 | 5’cgcgtaccctgacaccaatct3’ | 5’cagaagcagttccatttgca3’ | 387 |
| *Svs5* | NM_133516 | 5’-GTACCAGCAGCCATTTTGGT3’ | 5’GAACGAGGACAACCTGCCTA3’ | 154 |
| *Cdkn1a* | NM_080782 | 5’AGCAAAGTATGCCGTCGTCT3’ | 5’ACACGCTCCCAGACGTAGTT3’ | 150 |
| *Ptgds* | NM_013015 | 5’GGTTCCGGGAGAAGAAAGAG3’ | 5’CACTGAGAGGGAGTGGAAGC3’ | 200 |
| *Cxcl12* | NM_022177 | 5’-GTTTGCTTTGGAGCTTCTCG-3’ | 5’-GCTCTGGTGGAAGGTTGCTA-3’ | 99 |
| *Rps16* | X17665 | 5’AAGTCTTCGGACGCAAGAAA3’ | 5’TGCCCAGAAGCAGAACAG3’ | 146 |
