# Supplementary material for: Androgen and Luteinizing Hormone Stimulate the Function of Rat Immature Leydig Cells Through Different Transcription Signals
Source: Front Endocrinol (Lausanne). 2021 Mar 17;12:599149. doi: 10.3389/fendo.2021.599149 (PMC8011569; doi:10.3389/fendo.2021.599149)
Supplement: Supplementary file 4 [file Table_2.doc]

**Supplementary Table S2. Gene expression in steroidogenic pathway**

| **Ontogeny** | **Gene symbol** | **Density** | | | |
| --- | --- | --- | --- | --- | --- |
|  |  | **Control** | **MENT** | **LH** | **LH+MENT** |
| **Steroidogenic pathway** |  |  |  |  |  |
| Scavenger receptor class B type 1 | Scarb1 | 143±23 ^a^ | 42±2 (-3) ^a^ | 620±62(+4.3) ^b^ | 374±21(+2.6) ^c^ |
| Steroidogenic acute regulatory protein | Star | 6596±608 ^a^ | 3850±417 (-1.5) ^a^ | 7238±333(+1.1) ^a^ | 9647±1687(+1.5) ^a^ |
| Cytochrome P450 cholesterol side chain cleavage | Cyp11a1 | 393±43^a^ | 326±44 (-0.2)^a^ | 2682±100(+6.8)^b^ | 2312±276(+5.9)^b^ |
| Ferredoxin 1 | Fdx1 | 5940 ±366 ^a^ | 5892±342(-0.1) ^a^ | 10391±1349(+1.7) ^b^ | 10309±769(+1.7) ^b^ |
| Adrenodoxin reductase | Fdxr | 194±8.6 ^a^ | 92±7 (-2) ^a^ | 144±43(-0.3) ^a^ | 163±42(-0.2) ^a^ |
| 3β-Hydroxysteroid dehydrogenase 1 | Hsd3b1 | 5798±345 ^ab^ | 3233±134 (-0.6) ^b^ | 5912±1030(-0.1) ^ab^ | 11817±3199(+2.0)^ac^ |
| Sulfotransferase family 1A member 1 | Sult1a1 | 2042±12 ^a^ | 1230±108 (-0.7) ^b^ | 575±82(-3.3)^c^ | 577±175(-3.3) ^c^ |
| Cytochrome P450 17α-hydroxylase | Cyp17a1 | 288±12 ^a^ | 1046±73 (+3.6)^b^ | 564±219(+2.0) ^ab^ | 538±76(+2.0) ^ab^ |
| Steroid 5α-reductase 1 | Srd5a1 | 103±10 ^a^ | 133±7 (+1.3) ^ab^ | 154±12(+1.5) ^ab^ | 247±65(+2.4)^bc^ |
| 17β-Hydroxysteroid dehydrogenase 4 | Hsd17b4 | 5282±388 ^a^ | 3958±179(-0.3) ^a^ | 4867±495(-0.1) ^a^ | 4688±797(-0.1) ^a^ |
| 17β-Hydroxysteroid dehydrogenase 8 | Hsd17b8 | 1687±139 ^a^ | 1846±122(+1.1) ^a^ | 1274±106(-0.2) ^a^ | 1991±394(+1.2) ^a^ |
| 3α-Hydroxysteroid dehydrogenase (LOC191574) | Akr1c14 | 1440±115 ^a^ | 1210±65(-0.2) ^a^ | 478±79(-3.3) ^b^ | 736±98(-1.9) ^b^ |
| **Transcription factor** |  |  |  |  |  |
| Activating transcription factor 5 | Atf5 | 391±25 ^a^ | 1054±79(+2.7) ^b^ | 362±79(-0.1) ^a^ | 671±79(+1.7) ^c^ |
| Growth factor independent 1 transcriptional repressor | Gfi1 | 29±40 ^a^ | 156±6(+5.0) ^b^ | 73±22(+2.5) ^a^ | 72±21(+2.5) ^c^ |
| Pro-apoptotic WT1 regulator | Pawr | 1183±136 ^a^ | 3062±194(+2.6) ^b^ | 1999±242(+1.7) ^ab^ | 1548±495(+1.3) ^a^ |
| Peroxisome proliferator activated receptor delta | Ppard | 8±0.4 ^a^ | 53±1(+6.9) ^b^ | 18±4(+2.4) ^a^ | 31±16(+4.1) ^a^ |
| Early growth response protein 1 | Egr1 | 1349±263 ^a^ | 1492±149(+1.1) ^b^ | 3805±383(+2.8) ^b^ | 1822±214(+1.3) ^a^ |
| cAMP responsive element modulator | Crem | 235±16 ^a^ | 427±9(+1.8) ^a^ | 626±106(+2.7) ^b^ | 678±31(+2.9) ^b^ |
| RUNX family transcription factor 1 | Runx1 | 119±20^a^ | 64±3(-1.8) ^a^ | 886±110(+7.5) ^b^ | 187±37(+1.6) ^a^ |
| Activating transcription factor 4 | Atf4 | 1902±87^a^ | 5427±424(+2.9) ^b^ | 2380±336(+1.3) ^a^ | 3867±992(+2.0) ^a^ |
| Cyclin L1 | Ccnl1 | 932±50^a^ | 2543±123(+2.7) ^b^ | 1555±103(+1.7) ^a^ | 2083±592(+2.2) ^a^ |
| DNA damage inducible transcript 3 | Ddit3 | 821±101^a^ | 3547±213(+4.3) ^b^ | 1057±112(+1.7) ^a^ | 2314±767(+2.8) ^ab^ |
| Period circadian protein homolog 1 | Per1 | 165±27 ^a^ | 402±17(+2.4) ^b^ | 271±42(+1.6) ^ab^ | 368±87 (+2.2) ^ab^ |
| Signal transducer and activator of transcription 5B | Stat5b | 287±7^a^ | 669±36(+2.2) ^b^ | 406±50(+1.4) ^ab^ | 628±90 (+2.3) ^b^ |
| Tribbles homolog 3 | Trib3 | 51±10^a^ | 201±10(+3.9) ^b^ | 97±11(+1.9 ^a^ | 233±50(+4.5) ^b^ |
| Activating transcription factor 3 | Atf3 | 66±9^a^ | 583±24(+8.8) ^a^ | 611±359(+9.2) ^a^ | 346±163 (+5.2) ^a^ |
| BTG anti-proliferation factor 2 | Btg2 | 423±77^a^ | 2116±98(+8.8) ^ab^ | 3114±711(+9.2)^b^ | 1553±691 (+5.2) ^ab^ |
| Notch receptor 4 | Notch4 | 49±9^a^ | 136±13(+2.8) ^a^ | 212±55(+4.3)^a^ | 177±80(+3.6) ^a^ |
| SWI/SNF-related subfamily D member 2 | Smarcd2 | 119±24^a^ | 17±6(-6.9)^b^ | 66±8(-1.8)^ab^ | 45±9(-2.6) ^b^ |
| Amino-terminal enhancer of split 2 | Aes2 | 3373±766 ^a^ | 2167±205(-1.6)^ab^ | 1515±29(-2.2)^b^ | 1691±42(-1.9) ^ab^ |
| Zinc finger and BTB domain containing 7A | Zbtb7a | 917±283 ^a^ | 423±21(-2.2)^a^ | 450±10(-2.0)^a^ | 437±28(-2.1) ^a^ |
| Interferon regulatory factor 7 | Irf7 | 1400±200 ^a^ | 552±25(-2.5)^b^ | 500±39(-2.8) ^b^ | 462±29(-3.0) ^b^ |
| Nuclear receptor subfamily 1 Group H member 3 | Nr1h3 | 213±26 ^a^ | 60±11(-3.6)^b^ | 65±14(-3.3) ^b^ | 78±19(-2.7) ^b^ |

(Negative = down-regulation; positive = up-regulation) Fold changes after MENT, LH or LH+MENT compared to control. Mean ± SE, n =3; Identical letters designate that there was no significant difference between two groups at P <0.05.
